# Clinical characteristics and cerebrospinal fluid parameters in patients with peripheral facial palsy caused by Lyme neuroborreliosis compared with facial palsy of unknown origin (Bell's palsy)

**DOI:** 10.1186/1471-2334-11-215

**Published:** 2011-08-10

**Authors:** Daniel Bremell, Lars Hagberg

**Affiliations:** 1 Department of Infectious Diseases, Sahlgrenska University Hospital, 416 85 Gothenburg, Sweden

## Abstract

**Background:**

Bell's palsy and Lyme neuroborreliosis are the two most common diagnoses in patients with peripheral facial palsy in areas endemic for *Borrelia burgdorferi*. Bell's palsy is treated with corticosteroids, while Lyme neuroborreliosis is treated with antibiotics. The diagnosis of Lyme neuroborreliosis relies on the detection of *Borrelia *antibodies in blood and/or cerebrospinal fluid, which is time consuming. In this study, we retrospectively analysed clinical and cerebrospinal fluid parameters in well-characterised patient material with peripheral facial palsy caused by Lyme neuroborreliosis or Bell's palsy, in order to obtain a working diagnosis and basis for treatment decisions in the acute stage.

**Methods:**

Hospital records from the Department of Infectious Diseases, Sahlgrenska University Hospital, for patients with peripheral facial palsy that had undergone lumbar puncture, were reviewed. Patients were classified as Bell's palsy, definite Lyme neuroborreliosis, or possible Lyme neuroborreliosis, on the basis of the presence of *Borrelia *antibodies in serum and cerebrospinal fluid and preceding erythema migrans.

**Results:**

One hundred and two patients were analysed; 51 were classified as Bell's palsy, 34 as definite Lyme neuroborreliosis and 17 as possible Lyme neuroborreliosis. Patients with definite Lyme neuroborreliosis fell ill during the second half of the year, with a peak in August, whereas patients with Bell's palsy fell ill in a more evenly distributed manner over the year. Patients with definite Lyme neuroborreliosis had significantly more neurological symptoms outside the paretic area of the face and significantly higher levels of mononuclear cells and albumin in their cerebrospinal fluid. A reported history of tick bite was uncommon in both groups.

**Conclusions:**

We found that the time of the year, associated neurological symptoms and mononuclear pleocytosis were strong predictive factors for Lyme neuroborreliosis as a cause of peripheral facial palsy in an area endemic for *Borrelia*. For these patients, we suggest that ex juvantibus treatment with oral doxycycline should be preferred to early corticosteroid treatment.

## Background

Peripheral facial palsy occurs in the general population, with an annual incidence of 20-53 per 100,000 [1,2]. In areas endemic for *Borrelia burgdorferi *(*Bb*), Lyme neuroborreliosis (LNB) is estimated to cause 2-25% of peripheral facial palsy cases [3-6]. The remaining cases are caused by a wide range of diagnoses, such as Ramsay Hunt syndrome, sarcoidosis, Sjögren's syndrome, tumours and acute idiopathic peripheral facial palsy, also known as Bell's palsy (BP). Of these, BP constitutes by far the largest group, causing 60-75% of cases of peripheral facial palsy [2,7]. While LNB is treated with oral doxycycline or intravenous ceftriaxone, early treatment (within 72 hours) with corticosteroids improves the outcome in BP [8-12]. In order to choose the right treatment, it is important to differentiate between these two conditions. Antibodies to *Bb *in serum and cerebrospinal fluid (CSF) are often helpful in the diagnosis, but it generally takes a couple of days to obtain the analysis results. Furthermore, no data are available regarding the optimal treatment of patients with BP who present more than 72 hours after the onset of symptoms [8]. At the time of admission, the treatment decision must therefore frequently be based on patient history, physical examination and cerebrospinal fluid analysis of leukocytes, albumin and glucose, which can be obtained within hours. There is no time to wait for the results of other analyses. The aim of this study was retrospectively to analyse clinical and CSF parameters in well-characterised patient material with LNB and BP, where an acute lumbar puncture had been performed, in order to obtain a base for treatment decisions.

## Methods

### Patients

Hospital records for all the patients that presented at, or were referred to, the Department of Infectious Diseases, Sahlgrenska University Hospital, Gothenburg, Sweden, with peripheral facial palsy and in whom a lumbar puncture had been performed, between February 2000 and February 2009, were reviewed. Data on specific medical history, clinical characteristics and laboratory parameters were collected. Patients with peripheral facial palsy with causes other than LNB or BP were excluded.

### Case definitions

Patients were classified as BP, definite LNB, or possible LNB. Patients with *Bb *antibodies below the upper reference level in both serum and CSF, and with no history of erythema migrans (EM) within 3 months before the onset of neurological symptoms and with no other causes of peripheral facial palsy, were classified as BP. Patients with *Bb *antibodies (IgG and/or IgM) above the upper reference level in CSF and either a positive *Bb *antibody index or the presence of ≥ 2 oligoclonal bands on isoelectric focusing of CSF and serum, or with a history of EM within 3 months before the onset of neurological symptoms, were classified as definite LNB. Patients with *Bb *antibodies above the upper reference level in CSF and/or serum but with a negative *Bb *antibody index and < 2 oligoclonal bands on isoelectric focusing of CSF and serum and with no history of EM within 3 months before the onset of neurological symptoms were classified as possible LNB. The *Bb *antibody index was calculated as the ratio of the CSF/serum quotient of specific antibodies to the corresponding CSF/serum quotient of total immunoglobulins. Antibody index values of > 1.4 were considered positive [13].

For clarity, comparisons of clinical characteristics and laboratory parameters were made mainly between the definite LNB and BP groups, with the possible LNB group described in more detail separately. CSF pleocytosis was deliberately not used as a diagnostic criterion, since one aim of the study was to analyse differences in CSF parameters between the groups.

A subgroup analysis was made of patients with peripheral facial palsy without associated neurological symptoms.

### Laboratory tests

Two different tests were used to analyse *Bb *antibodies in serum and CSF during the study period. Until 26 June 2006, antibodies were analysed using an enzyme-linked immunosorbent assay (ELISA) kit for IgG and IgM antibodies (Dako Lyme Borreliosis Kit, Dako). Tests positive for IgM were further analysed with a more specific test (IDEIA, Dako). After 26 June 2006, *Bb *antibodies were analysed using a sandwich chemiluminescence immunoassay (CLIA) test kit (LIASON, DiaSorin).

### Statistics

Results were analysed using a statistical software package (PASW Statistics 18.0). For the analysis of unpaired categorical data, Pearson's χ^2 ^test was used. As the quantitative measures showed a markedly skewed distribution, they were analysed using the non-parametric Mann-Whitney *U *test. Quantitative variables are presented as the median (range). p-values of < 0.05 were considered statistically significant.

### Ethics

The study was approved by the Regional Ethical Review Board, Gothenburg, Sweden.

## Results

Hospital records for 109 patients were reviewed. Seven patients diagnosed with diseases other than LNB or BP were excluded (5 patients with Ramsay Hunt syndrome, 1 with brain stem infarction, 1 with erysipelas). In all, 102 patients were analysed; 51 were classified as BP, 34 as definite LNB and 17 as possible LNB. Of the patients diagnosed as definite LNB, 30 had a positive *Bb *antibody index. Of the remaining 4 patients, 2 had ≥ 2 oligoclonal bands (IgG or IgM) on isoelectric focusing of CSF and serum, while 2 had a preceding EM.

**Figure 1:**
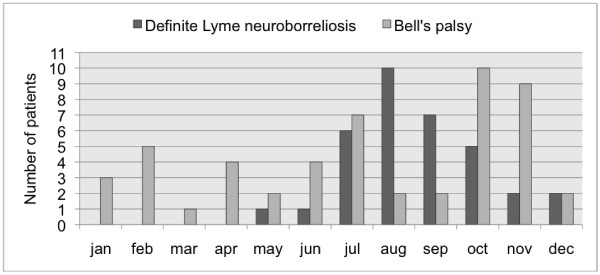
**Month of onset of symptoms**. Number of patients per month presenting with peripheral facial palsy for the groups Bell's palsy and definite Lyme neuroborreliosis.

Patients with definite LNB were older than patients with BP, median age 46 (range 7-75) and 36 (range 15-70) respectively, and fell ill during the second half of the year, with a peak in August, whereas patients with Bell's palsy fell ill in a more evenly distributed manner over the year (Figure 1). Patients with definite LNB more commonly had neurological symptoms outside the paretic area of the face than patients with BP; these symptoms included radiculitic pain (50% and 10% respectively, p < 0.001), sensibility disturbances (24% and 0%, p < 0.001) and pareses (9% and 0%, p = 0.03). Symptoms from the affected side of the face were equally common in both the definite LNB and BP group; pain (50% and 33%) and sensibility disturbances (6% and 12%). A reported history of tick bite within 3 months before the onset of symptoms was uncommon in both groups and there was no significant difference, 29% of definite LNB patients, 14% of BP patients (p = 0.08) (Table 1).

**Table 1:** Baseline data, clinical characteristics and CSF laboratory data

	Bell's palsy (n = 51)	Definite Lyme neuroborreliosis (n = 34)	Possible Lyme neuroborreliosis (n = 17)	p*
Gender, female	26 (51%)	13 (38%)	7 (41%)	

Age	36 (15-70)	46 (7-75)	28 (8-70)	*0.04*

Tick bite within 3 months	7 (14%)	10 (29%)	4 (24%)	*0.08*

Symptoms				

Local pain, affected side of face	17 (33%)	17 (50%)	7 (41%)	*0.43*

Radiculitic pain, other location	5 (10%)	17 (50%)	1 (6%)	*< 0.001*

Local sensibility disturbances	6 (12%)	2 (6%)	2 (12%)	*0.36*

Other sensibility disturbances	0 (0%)	8 (24%)	1 (6%)	*< 0.001*

Other pareses	0 (0%)	3 (9%)	1 (6%)	*0.03*

No associated neurogical symptoms	25 (49%)	6 (18%)	7 (41%)	

Duration of symptoms before LP, days	5 (1-90)	14 (1-75)	12 (2-120)	

CSF laboratory data				

Mononuclear cells, no/μL	2 (0-534)	165 (32-1044)	3 (0-41)	*< 0.001*

Albumin, mg/L	196 (76-768)	840 (197-2850)	204 (66-774)	*< 0.001*

Comparison between patients with peripheral facial palsy diagnosed as Bell's palsy, definite Lyme neuroborreliosis or possible Lyme neuroborreliosis. Data for gender, history of tick bite and symptoms are presented as number of patients (percentage of total for the group). Data for numerical values are presented as median (range). *p-values refer to the comparison between the groups with Bell's palsy and definite Lyme neuroborreliosis.

All the patients in the definite LNB group had an elevated number of CSF mononuclear cells (ref. < 5/μL), compared with 20% in the BP group. The median CSF mononuclear cell count in the definite LNB group was 165/μL (32-1044), while it was 2/μL (0-534) in the BP group (p < 0.001). CSF albumin levels were also significantly higher in the definite LNB group, median 840 mg/L (197-2850), than in the Bell's palsy group, median 196 mg/L (76-768) (p < 0.001) (Table 1).

The patients in the possible LNB group did not differ significantly from the patients in the BP group in any of the clinical characteristics or laboratory parameters described above. However, they differed significantly from the definite LNB patients in neurological symptoms outside the affected area of the face, radiculitic pain (6% and 50% respectively, p = 0.006) and sensibility disturbances (6% and 24%, p = 0.03), and also in CSF laboratory parameters, mononuclear cells (median 3/μL [0-41] and median 165/μL [32-1044] respectively, p < 0.001), and albumin (median 204 mg/L [66-774] and 840 mg/L [197-2850], p < 0.001) (Table 1).

Thirty-eight patients had no associated neurological symptoms and were analysed as a subgroup; 25 patients with BP, 6 with definite LNB, 7 with possible LNB. Among these patients, the median CSF mononuclear cell count in the definite LNB group was 103/μL (40-1044), while it was 2/μL (0-36) in the BP group (p < 0.001). CSF albumin levels were also significantly higher in the definite LNB group, median 988 mg/L (444-1520), than in the Bell's palsy group, median 194 mg/L (107-569) (p < 0.001) (Table 2).

**Table 2:** Subgroup analysis of patients without associated neurological symptoms

	Bell's Palsy (n = 25)	Definite Lyme Neuroborreliosis (n = 6)	Possible Lyme Neuroborreliosis (n = 7)	p*
Gender, female	16 (64%)	1 (17%)	4 (57%)	

Age	36 (15-70)	33 (19-67)	27 (8-70)	*0.84*

Tick bite within 3 months	2 (8%)	3 (50%)	3 (43%)	*0.12*

Duration of symptoms before LP, days	3 (1-25)	3 (1-42)	12 (2-120)	

CSF laboratory data				

Mononuclear cells, no/μL	2 (0-36)	103 (40-1044)	3 (1-14)	*< 0.001*

Albumin, mg/L	194 (107-569)	988 (444-1520)	175 (66-279)	*< 0.001*

Comparison between patients with peripheral facial palsy diagnosed as Bell's palsy, definite Lyme neuroborreliosis or possible Lyme neuroborreliosis and no associated neurological symptoms. Data for gender and history of tick bite are presented as number of patients (percentage of total for the group). Data for numerical values are presented as median (range). *p-values refer to the comparison between the groups with Bell's palsy and definite Lyme neuroborreliosis

## Discussion

Peripheral facial palsy of unknown origin (Bell's palsy) is a disease with a large number of differential diagnoses [14]. In our study, we required evidence of intrathecally produced antibodies or preceding erythema migrans for a definite LNB diagnosis. Intrathecal antibody production is generally used for a definite diagnosis, but it has low sensitivity in the very early phase of the disease [15]. In general, patients with LNB display intrathecally produced antibodies within two weeks of the onset of symptoms, but in some patients antibody production may be delayed for up to six weeks, which poses a diagnostic problem [16,17]. Furthermore, both the culture of CSF for *Bb *and the detection of *Bb *in CSF by specific PCR analysis have a very low sensitivity and are not used in clinical routine [18]. It is therefore difficult completely to rule out the possibility that some patients with LNB were classified as BP, especially since patients diagnosed as BP underwent LP earlier than patients diagnosed as LNB (definite or possible) (Table 1). However, clinical follow-up did not change the diagnosis in any patient with BP. With these diagnostic drawbacks, there will always be some patients for whom the diagnosis is difficult and who will be classified as unknown. On the other hand, using only serum antibodies against *Bb *as a diagnostic criterion will produce a large number of false positive cases, since the seroprevalence of antibodies against *Bb *is high in the general population; 7-29% in endemic areas of Sweden [19,20].

The possible LNB group in the study (who had *Bb *antibodies in serum but not in CSF and no history of EM) did not differ significantly from the BP patients in terms of clinical characteristics or laboratory parameters and did not, as a median, display mononuclear pleocytosis or elevated albumin in CSF. We interpreted this as a reflection of the high seroprevalence of *Bb *antibodies in the population, rather than a *Bb *infection as the aetiology of peripheral facial palsy.

Among the 38 patients without associated neurological symptoms that were analysed as a subgroup, BP patients, as expected, constituted the majority, 66% (n = 25), and patients with possible LNB 18% (n = 7). However, 16% (n = 6) of these patients were diagnosed as definite LNB, showing that the absence of associated neurological symptoms does not exclude LNB as a cause of peripheral facial palsy. The differences in the CSF mononuclear cell count and albumin levels were also significant in the subgroup analysis, underlining the importance of CSF sampling in the diagnosis of LNB.

The observed seasonal variation in the incidence of peripheral facial palsy caused by LNB, with a peak in late summer/early autumn and no cases during late winter/early spring, confirms previous studies and co-varies with the tick-feeding season. The absence of any cases outside that season is also evidence that the incubation period for LNB is rarely more than a couple of weeks, as previously described [3,5,6,21].

The differing clinical picture between peripheral facial palsy caused by LNB and BP, with a higher occurrence of neurological symptoms outside the cranial nerve area in peripheral facial palsy caused by LNB, is broadly in accordance with previous studies. Two small, early studies describe polyneuropathy and radiculitic pain as symptoms that predict LNB, whereas a larger study from 2002 describes headache, arthralgia and enlarged cervical lymph nodes [3,4,6]. What is not usually described, however, is the high percentage of patients with BP that describe pain from the affected area of the face (43%).

A reported history of tick bite was uncommon in the definite LNB group (29%). The same low numbers have also been seen in some earlier studies, while other studies have suggested the use of a history of tick bite in discriminating between BP and LNB [5,22,23]. It is therefore important to stress that the absence of a tick bite history does not exclude LNB.

The present study was performed at a department, which mainly treats adult patients. Consequently, the age of the patients in this study is not fully representative of all patients with BP or LNB. However, the median ages of the respective groups are similar to those reported previously. Patients with definite LNB were older than patients with BP, median age 46 (7-75) and 36 (15-70) respectively. This is in line with the bimodal age distribution of LNB, with a median in the fifth decade of life and with the reported peak incidence of BP around the second to fifth decade of life [14,24].

One aim of this study was to analyse differences in CSF parameters between the definite LNB and BP patient groups. It was therefore not possible to use CSF mononuclear pleocytosis as a diagnostic criterion for LNB. In spite of this, we found that time during the year, presence of neurological symptoms outside the affected area of the face and mononuclear pleocytosis in CSF were strong predictive factors for LNB. All these data are possible to obtain within hours after admission, which can be of help in the choice of acute treatment.

## Conclusions

We found that the time of the year, associated neurological symptoms and mononuclear pleocytosis in CSF were strong predictive factors for LNB as a cause of peripheral facial palsy in an area endemic for borreliosis. For these patients, we suggest that ex juvantibus treatment with oral doxycycline should be preferred to early corticosteroid treatment.

## Competing interests

The authors declare that they have no competing interests.

## Authors' contributions

DB and LH designed the study, carried out data collection and statistical analysis and drafted the paper. Both authors read and approved the final manuscript.

## Pre-publication history

The pre-publication history for this paper can be accessed here:

http://www.biomedcentral.com/1471-2334/11/215/prepub
